# The interplay of CD150 and CD180 receptor pathways contribute to the pathobiology of chronic lymphocytic leukemia B cells by selective inhibition of Akt and MAPK signaling

**DOI:** 10.1371/journal.pone.0185940

**Published:** 2017-10-05

**Authors:** Inna Gordiienko, Larysa Shlapatska, Valeriia Kholodniuk, Lilia Sklyarenko, Daniel F. Gluzman, Edward A. Clark, Svetlana P. Sidorenko

**Affiliations:** 1 Department of Molecular and Cellular Pathobiology, R.E. Kavetsky Institute of Experimental Pathology, Oncology and Radiobiology National Academy of Sciences of Ukraine, Kyiv, Ukraine; 2 Department of Oncohematology, R.E. Kavetsky Institute of Experimental Pathology, Oncology and Radiobiology National Academy of Sciences of Ukraine, Kyiv, Ukraine; 3 Department of Immunology, University of Washington, Seattle, Washington, United States of America; University of Manitoba, CANADA

## Abstract

Cell surface expression of CD150 and CD180 receptors in chronic lymphocytic leukemia (CLL) associates with mutational *IGHV* status and favourable prognosis. Here we show a direct correlation between cell surface expression and colocalization of these receptors on CLL B cells. In the absence of CD150 and CD180 on the cell surface both receptors were expressed in the cytoplasm. The CD150 receptor was colocalized with markers of the endoplasmic reticulum, the Golgi apparatus and early endosomes. In contrast, CD180 was detected preferentially in early endosomes. Analysis of CD150 isoforms differential expression revealed that regardless of CD150 cell surface expression the mCD150 isoform with two ITSM signaling motifs was a predominant CD150 isoform in CLL B cells. The majority of CLL cases had significantly elevated expression level of the soluble sCD150, moreover CLL B cells secrete this isoform. CD150 or CD180 crosslinking on CLL B cells alone led to activation of Akt, mTORC1, ERK1/2, p38MAPK and JNK1/2 networks. Both CD150 and CD180 target the translation machinery through mTOR independent as well as mTOR dependent pathways. Moreover, both these receptors transmit pro-survival signals via Akt-mediated inhibition of GSK3β and FOXO1/FOXO3a. Unexpectedly, coligation CD150 and CD180 receptors on CLL B cells led to mutual inhibition of the Akt and MAPK pathways. While CD150 and CD180 coligation resulted in reduced phosphorylation of Akt, ERK1/2, c-Jun, RSK, p70S6K, S6RP, and 4E-BP; it led to complete blocking of mTOR and p38MAPK phosphorylation. At the same time coligation of CD150 and CD40 receptors did not result in Akt and MAPK inhibition. This suggests that combination of signals via CD150 and CD180 leads to blocking of pro-survival pathways that may be a restraining factor for neoplastic CLL B cells propagation in more than 50% of CLL cases where these receptors are coexpressed.

## Introduction

Chronic lymphocytic leukemia (CLL) is the most common form of adult leukemia in Europe and North America [1]. A key feature of CLL is its extremely variable clinical outcome. Diverse genetic and epigenetic lesions, different phenotype profile and functional status of signaling molecules in malignant CLL B cells are molecular underpinnings of disease heterogeneity [2–6]. The main contributors to CLL pathogenesis are 1) antigenic B cell receptor (BCR) stimulation (microbial and autoantigens, neo-antigens created during apoptosis, autonomous signaling), 2) mutational status of the variable region of the immunoglobulin heavy (H) chain (*IGHV*) genes determining different response of malignant B cells to antigen stimulation, 3) involvement of different receptors in cell activation and interactions with the microenvironment, 4) genetic and epigenetic aberrations [1]. The dominant role in CLL pathogenesis belongs to BCR signaling. Indeed, targeted therapy directed against the BTK and PI3Kδ kinases, which are involved in BCR signaling, results in objective responses in more than 70% CLL patients [7,8]. Moreover, the BCR mutational status is one of the strongest predictors of disease outcome with unmutated *IGHV* CLL cases having a poorer prognosis [9]. In addition, high expression levels of CD38, CD49d and Zap70 in CLLs may serve as surrogate prognostic markers of unfavourable prognosis. CD38, CD49d and Zap70 directly or indirectly are involved in enhanced BCR signaling that leads to CLL B cells survival and proliferation [10].

The CD150 (IPO3/SLAM/SLAMF1) receptor is an adhesion and costimulatory molecule that may be involved in the regulation of CLL B cell microenvironment and pathobiology. CD150 is a multifunctional type I transmembrane glycoprotein that belongs to the SLAM family within the immunoglobulin superfamily of surface receptors [11–13]. It functions as a costimulatory molecule, a receptor for morbilliviruses, including measles virus, and also serves as bacterial sensor on macrophages [14–16]. Furthermore, CD150 cell surface expression on CLL B cells strongly correlates with mutated *IGHV* status and favourable clinical outcome [6,17,18]. CLL patients with CD150^+^ malignant B cells have longer treatment free and overall survival, compared to patients with CD150^-^ leukemic cells [18]. Thus, CD150 cell surface expression is a potential surrogate prognostic marker of CLL favourable outcome. Several alternatively spliced isoforms have been reported for CD150: the canonical transmembrane CD150 isoform (mCD150) with two ITSM signaling motifs in the cytoplasmic domain, a secreted CD150 isoform (sCD150) without a transmembrane region, and a novel CD150 isoform (nCD150) with an alternative cytoplasmic tail [19,20]. However, the profile of CD150 isoform expression in CLL has not been analysed.

CD180 is another putative surrogate marker for CLL favourable prognosis [21]. It is a pattern recognition receptor that regulates members of the Toll-like receptor (TLR) family and is involved in activation and proliferation of normal B cells [22–24]. Cell surface CD180 expression was detected in 60% of CLL cases [21]. However, the possible roles of the CD150 and CD180 receptors in CLL pathogenesis are not clear.

In the present study we show that CD150 and CD180 receptors are coexpressed and colocalized on the cell surface of CLL B cells. Moreover, in the absence of CD150 and CD180 on the cell surface both receptors were detected in the cytoplasm of malignant B cells. Our findings demonstrate that CLL B cells preferentially express the mCD150 isoform with elevated levels of soluble sCD150 isoform that lacks the transmembrane domain. We addressed the question whether simultaneous expression and cell surface colocalization of CD150 and CD180 might have any biological significance in CLL B cells pathobiology. In contrast to CD150 or CD180 ligation alone and CD150 and CD40 coligation, the combination of CD150 and CD180 crosslinking led to specific mutual inhibition of Akt and MAPK pathways affecting translational machinery and disrupting the propagation of pro-survival signals in CLL B cells.

## Materials and methods

### Patients

Blood samples from 67 previously untreated patients with verified CLL diagnosis were obtained from the Department of Oncohematology of R.E. Kavetsky Institute of Experimental Pathology, Oncology and Radiobiology of National Academy of Sciences of Ukraine (IEPOR NASU, Kyiv, Ukraine) according to the Institutional Review Board and Research Ethics Committees of IEPOR NASU. Written informed consent from each patient was obtained, and all experimental procedures were performed in accordance with the Declaration of Helsinki. Peripheral blood samples from healthy blood donors (HBD) were obtained from the Blood Transfusion Centre (Kyiv, Ukraine). Clinicopathological details of CLL cases are summarized in S1 Table.

### Antibodies

The list of followed antibodies against cell surface antigens were used for cell signaling, flow cytometry and confocal microscopy studies: mouse anti-CD20 (93-1B3), anti-CD22 (MYG13), anti-CD37 (IPO-24), anti-CD38 (AT1), anti-CD45 (Bra55), anti-CD48 (156-4H9), anti-CD95 (IPO-4), anti-CD150 (IPO-3) and anti-HLA-DR (IPO-10) (all from IEPOR NASU), goat anti-IgM (DAKO, Denmark), mouse anti-CD5 (10.2), anti-CD19 (HD37), anti-CD40 (G28-5), anti-CD180 (G28-8), and anti-MOPC21 (IgG1 isotype control) (University of Washington, Seattle, WA, USA). Primary rabbit antibodies against GRP78 (Tebu-bio, France), LAMP1 (Abcam, UK), TGN38 and EEA1 (both from Santa Cruz Biotech, USA) were used for visualisation of intracellular compartments. Secondary FITC-conjugated goat anti-mouse polyvalent IgG (Sigma-Aldrich, USA) was used for flow cytometry immunophenotyping, secondary goat anti-mouse IgG antisera Alexa Fluor 594 conjugate and goat anti-rabbit IgG antisera Alexa Fluor 546 conjugate (Molecular Probes, Thermo Fisher Scientific, USA) were applied for confocal microscopy. Antibodies used for western blot analysis included rabbit anti-pAkt (S473), anti-pAkt (T308), anti-pERK1/2 (T202/Y204), anti-pp38MAPK (T180/Y182), anti-pJNK1/2 (T183/Y185), anti-pc-Jun (S73), anti-p4E-BP1 (T37/46), anti-pp70S6K (T389), anti-pS6 Ribosomal Protein (S235/236), anti-pGSK3β (S9), anti-pmTOR (S2448), anti-pFOXO1(T24)/FOXO3a(T32), phospho-(S/T) Kinase Substrate Antibody Sampler Kit (all from Cell Signaling Technology, Beverly, MA, USA), goat anti-actin, (Santa Cruz Biotechnology, USA), monoclonal rabbit anti-CD150 (Sino Biologicals Inc., China), monoclonal mouse anti-pY (4G10 Sigma-Aldrich, USA). Secondary goat anti-rabbit and donkey anti-goat HRP-conjugated antibodies were from Santa Cruz Biotechnology (USA).

### Cell separation and stimulation

Peripheral blood mononuclear cells (PBMCs) from CLL patients and HBD were isolated by Lymphoprep (Axis-Shield PoCAS, Norway) density gradient centrifugation. PBMCs of HBD were depleted of T cells by E-rosetting with sheep red blood cells. Then, CD19^+^ and CD19^+^CD5^+^ subpopulations of normal B cells were isolated by magnetic separation using Dynabeads coated with sheep anti-mouse IgG (Dynal Biotech ASA, Norway) according to manufacturer’s protocol.

PBMCs from CLL patients were used for experiments only when level of CD19^+^ cells was >95% and no CD3^+^ cells expressed CD150. 10x10^6^ CLL PBMCs with the levels of CD150 and CD180 coexpression >20% were incubated with either anti-CD150 IPO-3 (10 μg/ml) alone, anti-CD180 (10 μg/ml) alone or a combination of these mAbs in RPMI-1640 medium supplemented with 10% FCS for signaling in CLL B cells. Stimulation of CD40 receptor was performed with anti-CD40 mAb in concentration 1 μg/ml. Stimulation was stopped after 5, 15 and 30 min of incubation with ice-cold PBS+0,1% NaN_3_.

### Flow cytometry

PBMCs from CLL patients were immunophenotyped using a standard indirect immunofluorescent method and analysed with EpicsXL fluorescent flow cytometer (Beckman Coulter, USA) using FCS Express V3 (De Novo Software). Results are presented as percentage of positive cells and GeoMean MFI ratio of antigen to isotype control, where 1–1.1 r.u. and ≤ 6% of positive cells in population correspond to negative cell surface expression of antigen, GeoMean ratio up to 2 r.u.–low cell surface expression, GeoMean ratio from 2 to 3 r.u.–medium expression, GeoMean ratio > 3 means high cell surface expression of antigens.

### Western blot analysis

Cell lysis, gel electrophoresis, western blot analysis were performed as described earlier [25]. The Microcon YM-3 (EMD Millipore, USA) were used for culture media concentration. Clarity Western ECL substrate (Immuno-Star HRP, BioRad, USA) and medical X-ray film (AGFA, Belgium) were used for visualization.

### qRT-PCR

Total RNA was isolated from cells using TRIzol reagent (Sigma-Aldrich, St. Louis, MO, USA) according to the manufacturer’s protocol. Two μg of isolated RNA were reverse transcribed to cDNA with RevertAid Reverse Transcriptase, RiboLock RNase Inhibitor, and Oligo(dT)_18_ anchored primer (all Thermo Scientific, USA). qRT-PCR was performed using Maxima SYBR Green/ROX qPCR Master Mix (Thermo Scientific, USA) on a sequence detection system 7500 (Applied Biosystems, CA, USA) and analyzed by 7500 System SDS Software (version 1.3.1). The sequence of primers used in this study and PCR cycling conditions are listed in S2 Table. Expression of the TATA-box binding protein (TBP) was used as an endogenous control for standardization. Ct values were determined for the internal control (TBP) and the test genes at the same threshold level in the exponential phase of the PCR curves. Relative quantification (comparative Ct (ddCt) method) was used to compare the expression level of the tested genes with the internal control and was represented in relative units. Dissociation curve analysis was performed after every run to check the specificity of the reaction. Three reactions (each in triplicate) were run for each gene, and the standard error of mean was calculated.

### Immunostaining and confocal microscopy

To differentiate cell surface and intracellular expression of CD150, CLL cells were resuspended in ice-cold PBS+2%FCS+0,1%NaN_3_ and attached to poly-L-lysine precoated microscope slides during 10 min at 4°C. Then, live cells were incubated for 1 hour with anti-CD150 mAb at 4°C, followed by secondary Alexa Fluor 594 goat anti-mouse IgG antibody (Molecular Probes, Invitrogene, USA) incubation for 30 min at 4°C and fixation/permeabilization in a mixture of cold methanol:acetone (1:1) for 1 hour at -20°C. After 1 hour rehydratation and blocking in 5% BSA samples were incubated with FITC-conjugated anti-CD150 mAb for 1 hour. Slides were washed three times and DAPI (1 μg/ml) was added for DNA staining.

To reveal the subcellular localisation of CD150 or CD180 in CLL B lymphocytes, cells were attached to microscope slides using a Cytospin centrifuge followed by methanol:acetone fixation/permeabilization, rehydratation and incubation with blocking solution (5% normal goat serum in PBS). The CLL cells were stained with primary rabbit antibodies to one of the intracellular compartments markers (EEA1, TGN38, LAMP1 or GRP78) for 1 hour, followed by secondary goat anti-rabbit conjugated with Alexa Fluor 546 plus anti-CD150 or anti-CD180 conjugated with FITC. Live cells of CD150 cell surface positive (csCD150^+^) CLL cases were stained with primary antibodies against cell surface receptors for 1 hour at 4°C. Secondary goat anti-mouse Ig linked with Alexa Fluor 594 or rabbit anti-goat Ig linked with Alexa Fluor 594 and anti-CD150-FITC were added to cells for 30 min at 4°C. Cells were fixed in 1% solution of paraformaldehyde.

The microscope slides were analysed using laser confocal scanning microscope LSM 510 META (Carl Zeiss, Germany) with next processing using Zeiss LMN Image Browser. Pearson’s correlation coefficient (R) and Manders coefficient (R[r]) of colocalization were measured using ImageJ software (MacBiophotonics, USA). Pearson’s coefficient (R) range is -1,0,1 where -1 means negative correlation between signals of two channels, 0 –absence of any correlation and 1 –is a strong positive correlation between signals of two channels. Manders overlap coefficient (R[r]) measured in range from 0 to 1, where 0 is defined as no colocalization and 1–100% of colocalization. Results were represented as mean±SEM.

### ELISA

Serum samples of health individuals and CLL patients were collected and frozen at -20°C. To evaluate levels of the sCD150 isoform in sera we used ELISA. The wells of 96-immunoplates were coated with serum samples diluted 1:10 in PBS overnight at 4°C. After washing with PBS+0,05% Tween-20 wells were blocked in 5% BSA during 2 hour at 37°C. Primary rabbit anti-CD150 antibody (Sino Biologicals Inc., China) was added to the wells in dilution 1:2000 for 1 hour at 37°C followed by secondary goat anti-rabbit HRP-conjugated antibodies (1:5000) (Santa Cruz Biotechnology, USA). Results were determined by o-phenylendiamine-containing substrate solution. The optical density was read at 490 nm. Reaction was performed in triplicates for each sample.

### Statistical analysis

Statistical significance between groups was assessed by unpaired Mann-Whitney U test. Cumulative results of experiments with stimulation and colocalization were presented as mean±SEM. Pearson’s coefficient was used for determination of correlation between variables. Results of CD150 mRNA expression were presented as box plots where whiskers means maximum and minimum values, the line within the rectangle shows the median, and the top and bottom of the rectangle represent the third and first quartile, respectively.

## Results

### CD150 is colocalized with CD180 on CLL B cells

**Fig 1:**
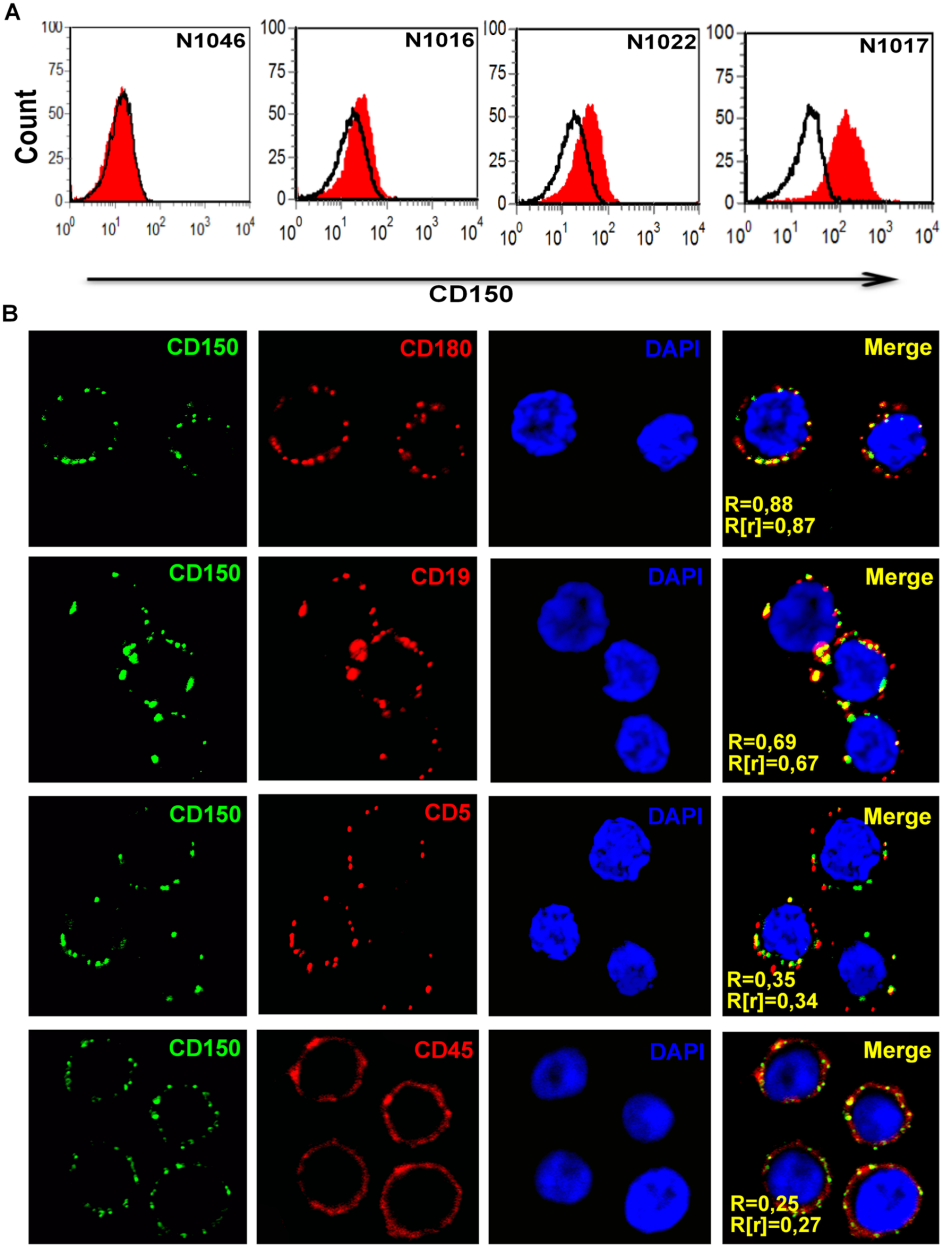
CD150 distribution on CLL B cells. (A) Flow cytometry analyses of cell surface CD150 (csCD150) expression on CLL B cells. CLL case N1046 represented a negative example of csCD150 expression (28.4% of cases), N1016 –low csCD150 expression (50.8% of cases), N1022 –medium csCD150 expression (10.4% of cases), and N1017 –high CD150 cell surface expression (10.4% of cases). (B) CD150 colocalization with receptors that are involved in modulation of BCR-mediated signaling in CLL B cells. Live cells were stained with primary antibodies (anti-CD180, CD19, CD5 or CD45) followed by secondary goat anti-mouse antibodies linked with Alexa Fluor 594 (red) and anti-CD150-FITC (green). Cells were fixed in a 1% solution of paraformaldehyde, and nuclei were stained with DAPI. Pearson’s correlation coefficient (R) and Manders coefficient (R[r]) of colocalization were measured using ImageJ software. CD150 had the highest level of colocalization with CD180. Representative results from three independent experiments. Microscopy magnification for all pictures was 630x.

Immunophenotyping of CLL cases showed typical expression of CD5, CD19, CD23 and CD43. In all examined CLL cases malignant B cells consistently expressed high levels of cell surface CD37, CD48 and CD40 (S3 Table). At the same time, there was significant heterogeneity within CLL cases in expression of CD150, CD180, CD20, CD22 and CD95 (Fig 1A, S3 Table). CD150 cell surface expression (csCD150) was detected in 71.6% of examined CLL cases with the portion of positive cells in the range from 7 to 93%. In 50.8% CLL cases B cells expressed low levels of csCD150. Medium expression level of CD150 was detected in 10.4% of CLL cases. Only 10.4% of CLL cases were characterised by high level of CD150 cell surface expression with more than 60% CD150^+^ CLL B cells. The expression of csCD150 positively correlated with the expression of the cell death receptor CD95 (r = 0.70, p = 0.0001) and CD180 (r = 0.75, p = 0.0001). It should be noted that in 59% of CLL cases B cells coexpress CD150 and CD180. Moreover, CD180 showed the highest level of colocalization with CD150 on the cell surface of CLL B cells (Fig 1B). CD150 was also colocalized with CD19 (R = 0.69±0.13; R[r] = 0.67±0.1), CD20 (R = 0.71±0.08; R[r] = 0.69±0.1), and CD37 (R = 0.67±0.15; R[r] = 0.65±0.12). At the same time we did not detect CD150 colocalization with either the BCR (R = 0.35±0.09; R[r] = 0.3±0.1), CD5 (R = 0.35±0.1; R[r] = 0.34±0.1), CD45 (R = 0.25±0.07; R[r] = 0.27±0.05), or CD40 (R = 0.41±0.15, R[r] = 0.42±0.2) (Fig 1B). Thus, CLL cases are heterogeneous in their CD150 cell surface expression. The csCD150 expression positively correlated with csCD180 and these receptors showed the highest level of colocalization on the plasma membrane of CLL B cells.

### Heterogeneity of CLL cases in CD150 topology

**Fig 2:**
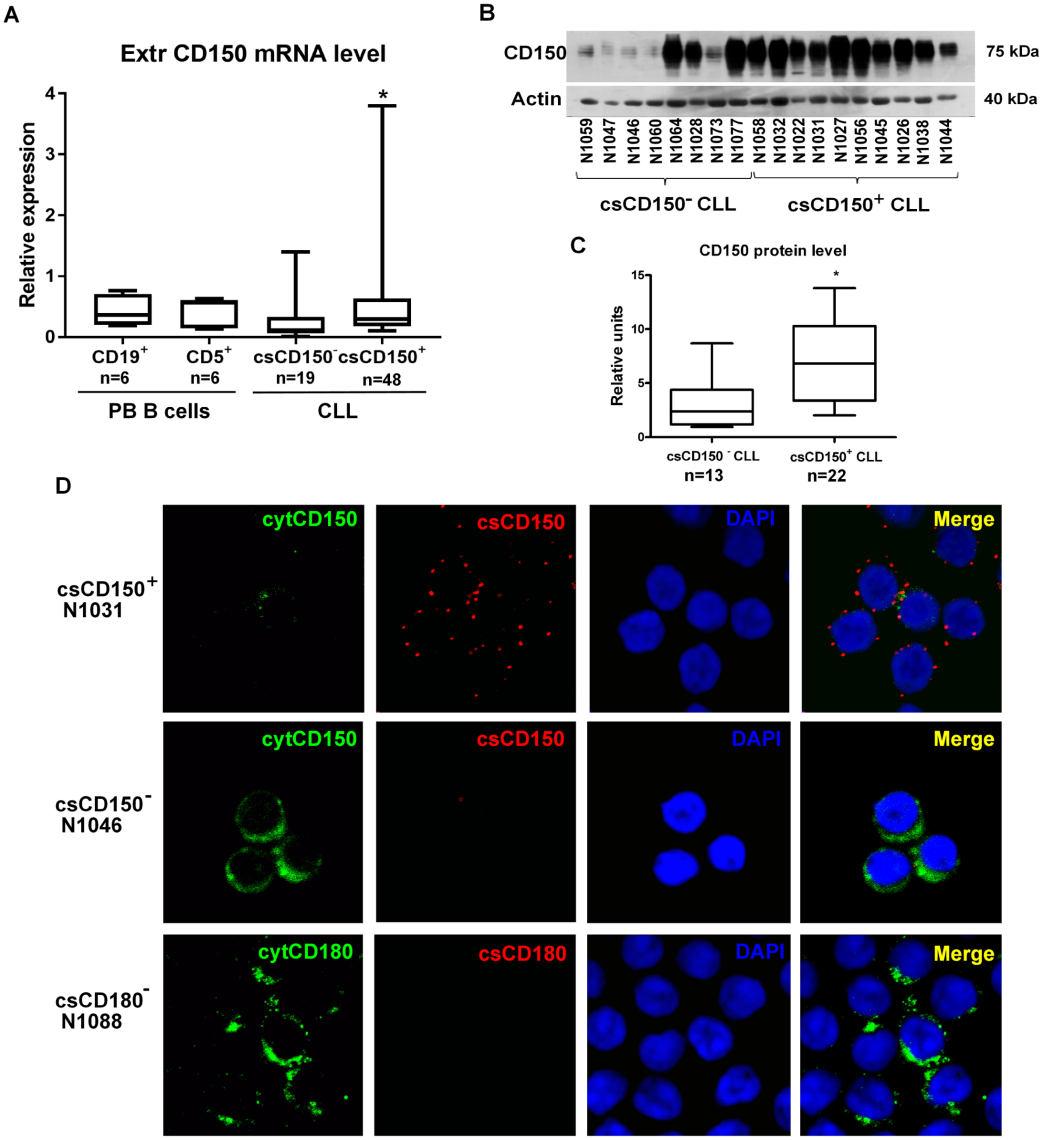
CD150 expression and topology in CLL B cells. (A) Total CD150 mRNA expression levels in normal peripheral blood CD19^+^ and CD5^+^ B cells, csCD150^-^ and csCD150^+^ CLL cases. Results of qRT-PCR analyses using primers specific for the region encoding the CD150 extracellular domain. Expression of CD150 was normalized to TBP expression level. Box plots showed quartiles, median, minimum and maximum values. * p = 0.01 compared to csCD150^-^ CLL cases. (B) Western blot analysis of CD150 expression in csCD150^-^ and csCD150^+^ CLL samples using a rabbit anti-CD150 mAb. One of three experiments is presented. (C) Densitometry analysis of CD150 protein expression in CLL cases. Results were normalized to actin expression. CD150 protein expression was detected in all csCD150^-^ CLL samples, but on significantly lower level than in csCD150^+^ CLL samples. * p = 0.05 compared to csCD150^-^ CLL cases. (D) Differential cell surface (cs) and cytoplasmic CD150 and CD180 expression in CLL B cells. Live cells were incubated with primary anti-CD150 or anti-CD180 mAbs followed by secondary Alexa Fluor 594 (red) goat anti-mouse antibody and fixation/permeabilization. Then samples were incubated with FITC conjugated anti-CD150 mAb (green) and DAPI was added for DNA staining. In csCD150^+^CLL samples CD150 was predominantly expressed on the cell surface (upper panel). In csCD150^-^ CLL samples CD150 was detected exclusively in the cytoplasm (middle panel). CD180 in csCD180^-^ CLL samples also was restricted to the cytoplasm (low panel). Confocal microscope magnification 630x. Representative results of five independent experiments.

We tested whether csCD150^-^ CLL B cells expressed CD150 at the mRNA level. To evaluate total CD150 mRNA levels, we used primers specific to the region encoding the extracellular part of CD150 that is common for all experimentally confirmed CD150 isoforms. Populations of CD19^+^ B cells and CD5^+^ B cells from the peripheral blood of healthy donors were used as normal counterparts of CLL malignant B cells. Total CD150 mRNA expression levels were slightly lower in CLL cases, compared to normal peripheral blood B-cell subsets (Fig 2A). csCD150^+^ CLL B cells expressed significantly higher CD150 mRNA levels than csCD150^-^ CLL B cells (p = 0.01). To address whether the CD150 mRNA in csCD150^-^ CLLs cases was translated into protein, we performed western blot analyses. CD150 protein was expressed in all tested CLL samples (Fig 2B and 2C). In about 45% of the csCD150^-^ samples CD150 protein expression was low, while in the remaining csCD150^-^ CLL cases, it was similar to levels in csCD150^+^ CLLs (Fig 2B and 2C). To clarify these results further, we used a specific immunostaining method followed by confocal microscopy visualization that allowed us to differentiate cell surface and intracellular CD150 expression (Fig 2D). In the csCD150^+^ CLLs CD150 was expressed predominantly on the cell surface and with only trace amount detected in the cytoplasm (Fig 2D, upper panel). In contrast, in the csCD150^-^ CLL cases CD150 was expressed exclusively in the cytoplasm (Fig 2D, middle panel). Cytoplasmic CD150 expression was found in all csCD150^-^ CLL cases in 32 to 90% malignant B cells. It is noteworthy that CD180 demonstrated a similar pattern of expression to that of CD150. In csCD180^-^ CLL cases intracellular CD180 expression was detected in 70–90% of malignant B cells (Fig 2D, lower panel).

**Fig 3:**
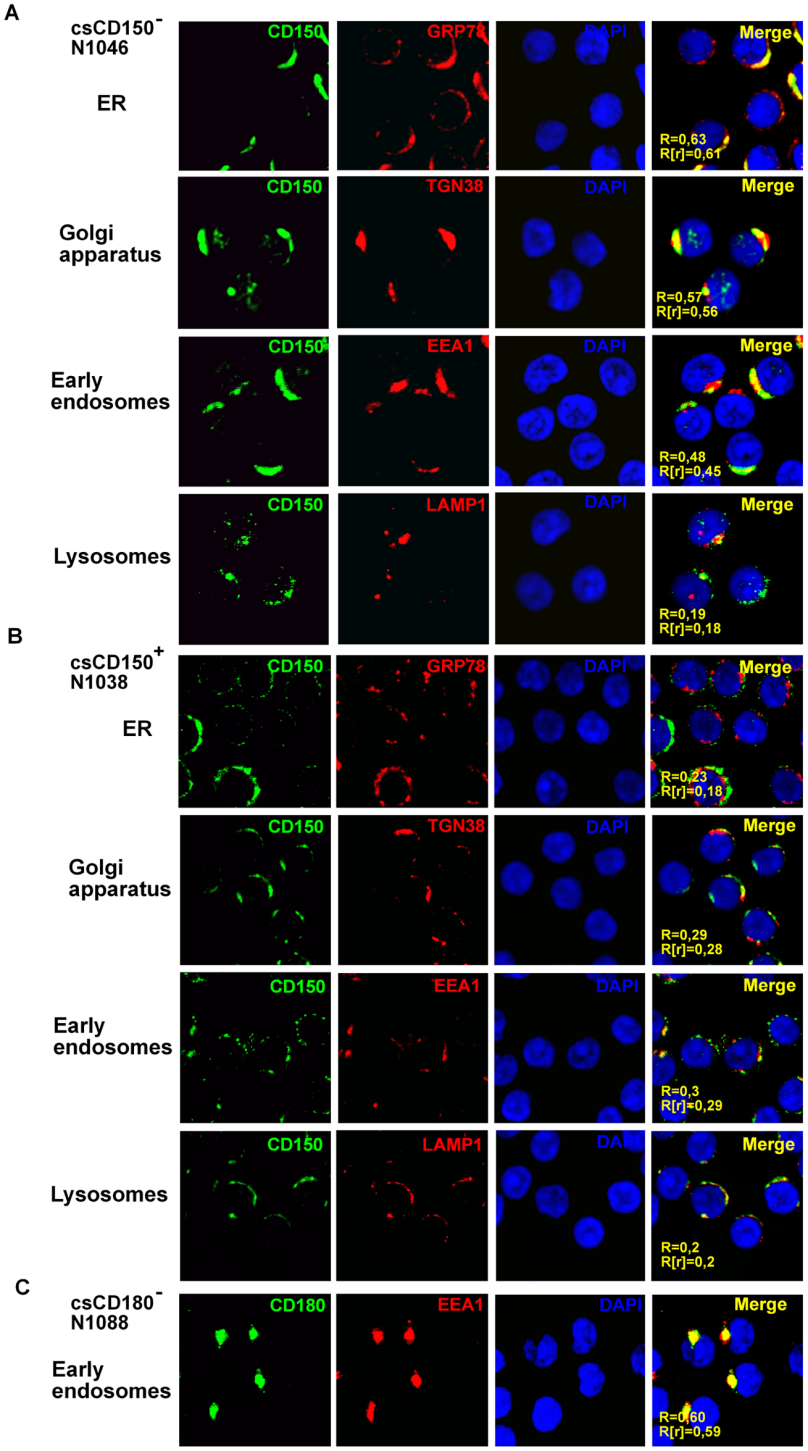
Intracellular localization of CD150 and CD180 in CLL B cells. Cells were fixed and simultaneously stained with anti-CD150 or anti-CD180 mAbs (green) and markers of the ER—GRP78; the Golgi apparatus—TGN38; early endosomes—EEA1; and lysosomes—LAMP1 (red). The level of colocalization with markers of intracellular compartments was defined by Pearson’s (R) and Manders (R[r]) coefficients using ImageJ software. (A) In csCD150^-^ CLL B cells CD150 was localized to ER, Golgi apparatus, early endosomes, but not to lysosomes. The results represent one of seven analyzed csCD150^-^ CLL cases. (B) In csCD150^+^ CLL B cells CD150 was mainly expressed on the cell surface with trace amounts in intracellular compartments. One of five analyzed csCD150^+^ CLL cases. (C) In csCD180^-^ B cells CD180 was accumulated in early endosomes. One of three analyzed csCD180^-^ CLL cases. Microscope magnification 630x.

To obtain further insight into the CD150 intracellular localization in CLL B cells, we performed simultaneous immunostaining of CD150 and markers of different intracellular compartments (Fig 3). In csCD150^-^ B cells a high level of CD150 colocalization was found with a marker of the endoplasmic reticulum (ER) GRP78 and a marker of the Golgi apparatus TGN38 (Fig 3A). Approximately 50% of intracellular CD150 was localized to early endosomes (EEA1), while only a trace amount of CD150 was detected in lysosomes (LAMP1) (Fig 3A). In contrast, in the csCD150^+^ CLL cases a low degree of CD150 colocalization was detected with markers of the ER (R = 0.23±0.05, R[r] = 0.19±0.10, n = 8), Golgi complex (R = 0.29±0.07, R[r] = 0.28±0.03, n = 8), endosomes (R = 0.30±0.03, R[r] = 0.29±0.05, n = 8) and lysosomes (R = 0.20±0.05, R[r] = 0.19±0.04, n = 8) (Fig 3B). This suggests that CD150 is mostly translocated to the plasma membrane in csCD150^+^ CLLs with a small quantity remaining in cytoplasm. However, in csCD150^-^ CLLs CD150 localizes just to intracellular compartments. In contrast to CD150, cytoplasmic CD180 in csCD180^-^ CLL cases was colocalized with a marker of early endosomes—EEA1 (Fig 3C), but had only a low degree of colocalization with markers of the ER—GRP78 (R = 0.24±0.05; R[r] = 0.20±0.05), Golgi apparatus—TGN38 (R = 0.23±0.04; R[r] = 0.22±0.03), and lysosomes—LAMP1 (R = 0.23±0.05; R[r] = 0.19±0.03).

Taken together, in all CLL cases CD150 was expressed at the protein level but with different topology. In csCD150^+^ CLL cases CD150 was mostly translocated to the plasma membrane with trace amounts remaining in the cytoplasm. The cell surface CD150 and CD180 double negative CLL B cells retained both receptors in cytoplasmic compartments.

### Differential expression of CD150 isoforms in CLL B cells

The restricted CD150 cytoplasmic expression in csCD150^-^ CLL B cells may be linked to the structural features of this receptor, as CD150 can be expressed in several alternatively spliced isoforms [19,20]. To find out which CD150 isoforms are expressed in CLL B cells, we focused on the canonical transmembrane CD150 isoform (mCD150) that has two ITSM signaling motifs in cytoplasmic domain, the secreted CD150 isoform (sCD150) that lacks the transmembrane region, and a novel CD150 isoform (nCD150) with an alternative cytoplasmic tail without known signaling motifs. To discriminate the mRNA expression of these CD150 isoforms we performed qRT-PCR using primers that are specific to unique region of each isoform (S2 Table).

**Fig 4:**
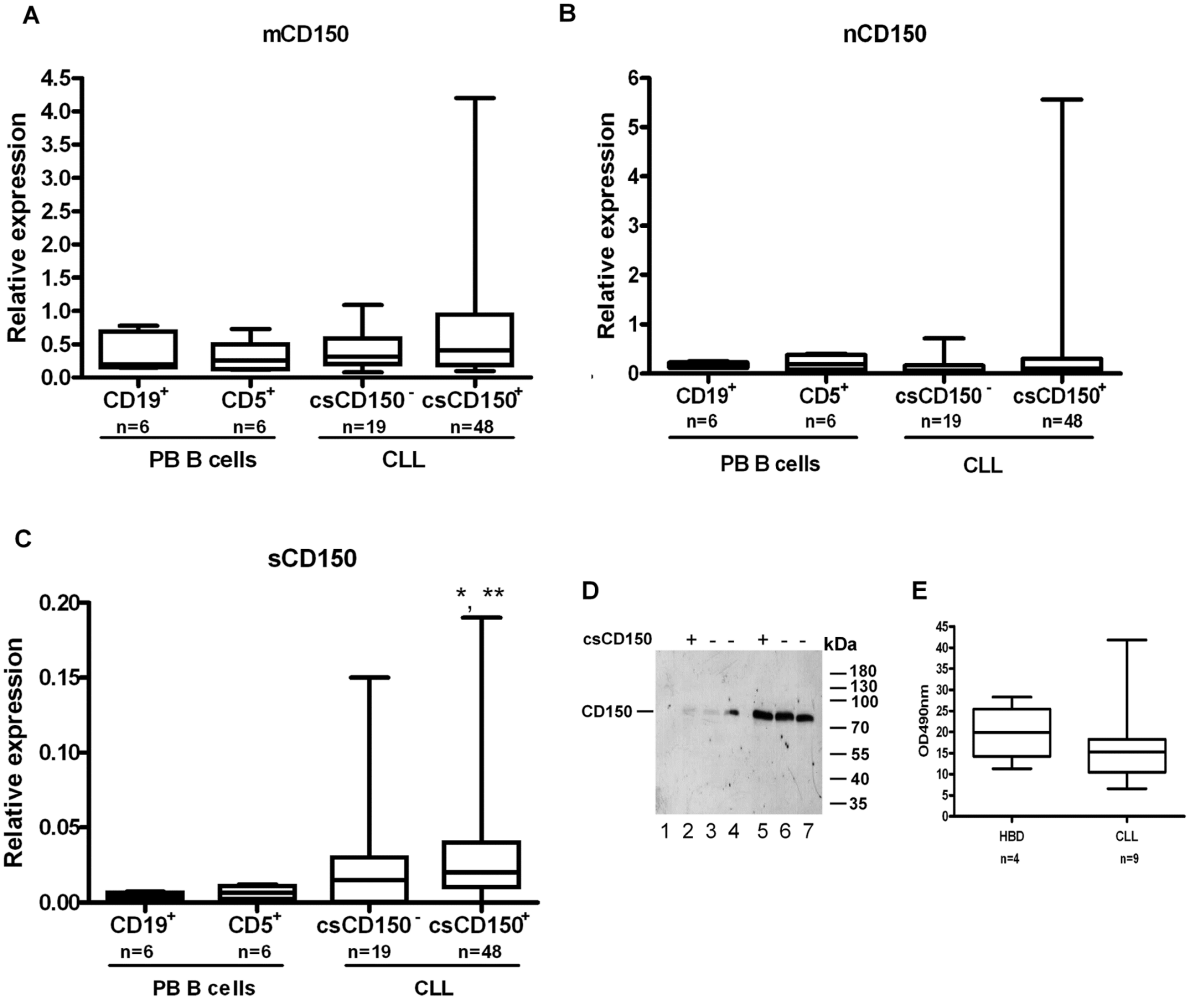
Expression of CD150 isoforms in CLL B cells. mRNA expression levels of CD150 isoforms were determined in normal CD19^+^ and CD5^+^ peripheral blood B cells and CLL samples that were grouped according to csCD150 expression: csCD150^-^ CLL cases (third column) and csCD150^+^ CLL cases with more than 6% of CD150^+^ cells (fourth column). (A) Expression levels of the transmembrane canonical mCD150 isoform. (B) Expression of nCD150 isoform with an alternative cytoplasmic tail. (C) Expression of soluble sCD150 isoform lacking a transmembrane region. * p<0.05 compared to CD19^+^ B cells, ** p<0.05 compared to CD5^+^ B cells. The qRT-PCR analysis. Expression of all isoforms was normalised to TBP expression levels. Box plots showed quartiles, median, minimum and maximum value. (D) CLL B cells secrete sCD150 in culture media. Western blot analysis of culture supernatants (tracks 2–4), 5x concentrate of culture supernatants (tracks 5–7) from 3 of 9 tested patients. Track 1 –control culture media. (E) The level of sCD150 in CLL patients’ blood sera, ELISA.

In the csCD150^+^ CLL samples mRNA expression level of the mCD150 isoform positively correlated with CD150 cell surface levels (r = 0.4, p<0.05) with the median of expression in the range of normal B cell subsets (Fig 4A). The median of mRNA expression of the nCD150 isoform in CLL B cells was slightly lower, compared to that in normal CD19^+^ or CD5^+^ B cells (Fig 4B). However, 15% of studied CLL cases were characterised by elevated nCD150 mRNA levels (up to 28 times higher than in normal counterparts) that did not correlate with CD150 cell surface expression (Fig 4B). Expression of sCD150 mRNA was higher in vast majority of studied CLL cases compared to normal B-cell subsets with significantly elevated sCD150 expression level in csCD150^+^ CLL cases (Fig 4C) (p<0.05). Indirect evidence of sCD150 protein expression in CLL samples was provided by western blot analyses (Fig 2B). There were visible three bands in CLL cases with low CD150 expression (N1059, N1047, N1046, N1060, N1073). The lowest band corresponded to the predicted size of sCD150, medium—mCD150 and the upper one—nCD150 isoform. To answer the question whether sCD150 could be secreted by CLL B cells, we cultured leukemic B cells for 48 hrs then did western blot analysis of culture supernatants (Fig 4D). Low levels of secreted sCD150 were detected in both csCD150 positive and negative cases (Fig 4D, tracks 2–4), and concentration of the culture supernatants (5x) allowed the specific bands to be detected better (Fig 4D, tracks 5–7). The sCD150 isoform was also detected in the sera of CLL patients; however we did not reveal significant differences with sCD150 level in healthy individuals (Fig 4E). Our results show that sCD150 isoform is expressed not only at the mRNA level, but also at the protein level and is actively secreted by CLL B cells. Thus, CD150 isoforms are differentially expressed in CLL B cells with the mCD150 isoform being the most prevalent. The presence of sCD150 protein could partially contribute to the restricted cytoplasmic CD150 expression in csCD150^-^ B cells.

### CD150 signaling in CLL B cells

**Fig 5:**
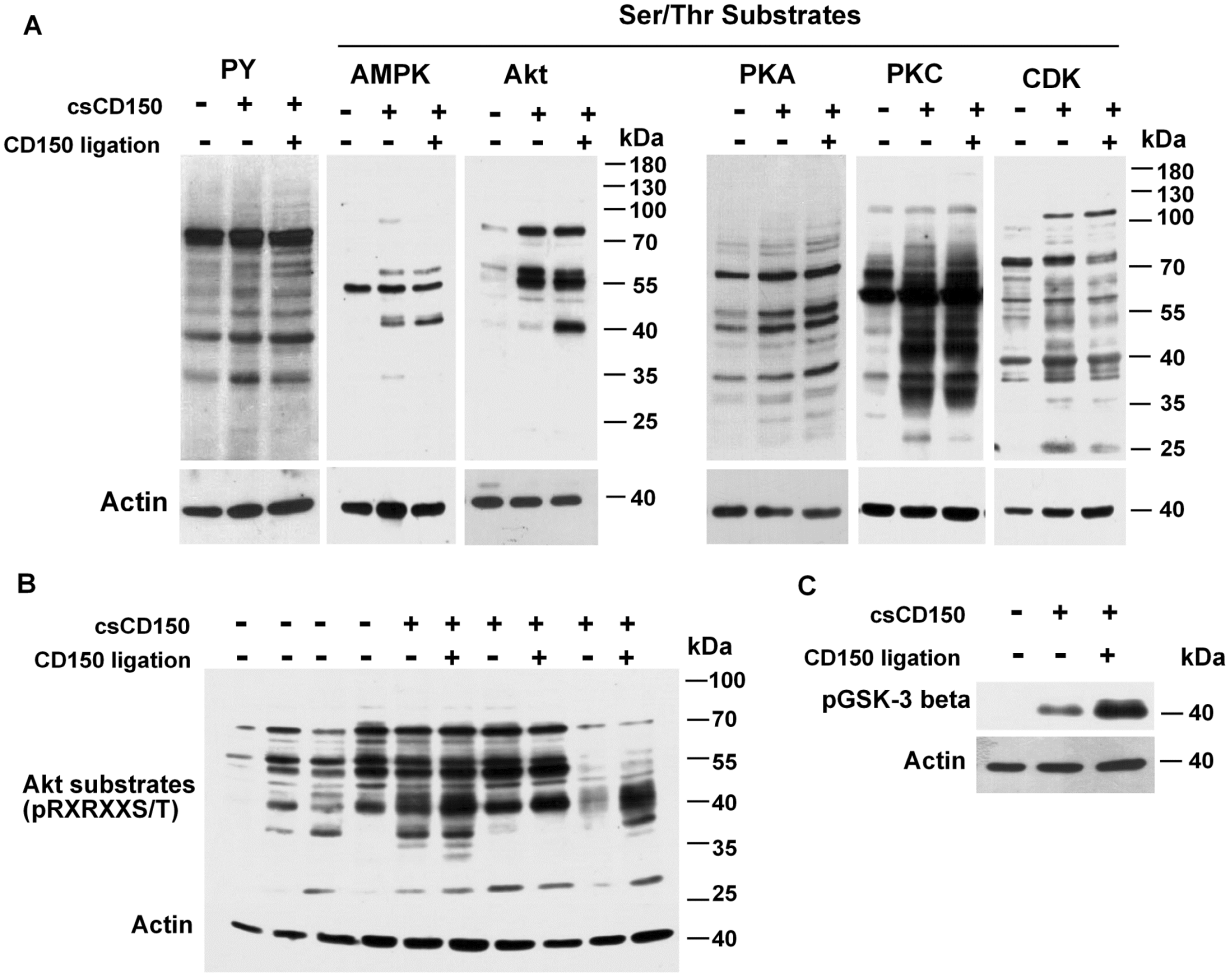
Phosporylation profile of csCD150^-^ and csCD150^+^ CLL cases. (A) Phosphorylation of tyrosine and AMPK, Akt, PKA, PKC, and CDK Ser/Thr substrates in csCD150^-^ (left columns), csCD150^+^ CLL cases (middle columns) and after CD150 ligation of csCD150^+^ CLLs (right columns). (B) Phosphorylation levels of Akt Ser/Thr motif (pRXRXXS/T) in csCD150^-^CLL cases, csCD150^+^ CLL cases and effect of CD150 ligation on Akt Ser/Thr motif phosphorylation. CD150 ligation resulted in increased phosphorylation of an Akt substrate with a molecular weight of about 40kDa. (C) CD150 ligation led to increased GSK3β phosphorylation in CD150^+^ CLLs. One of three independent experiments with different csCD150^-^ and csCD150^+^ CLL cases. Western blot analysis.

**Fig 6:**
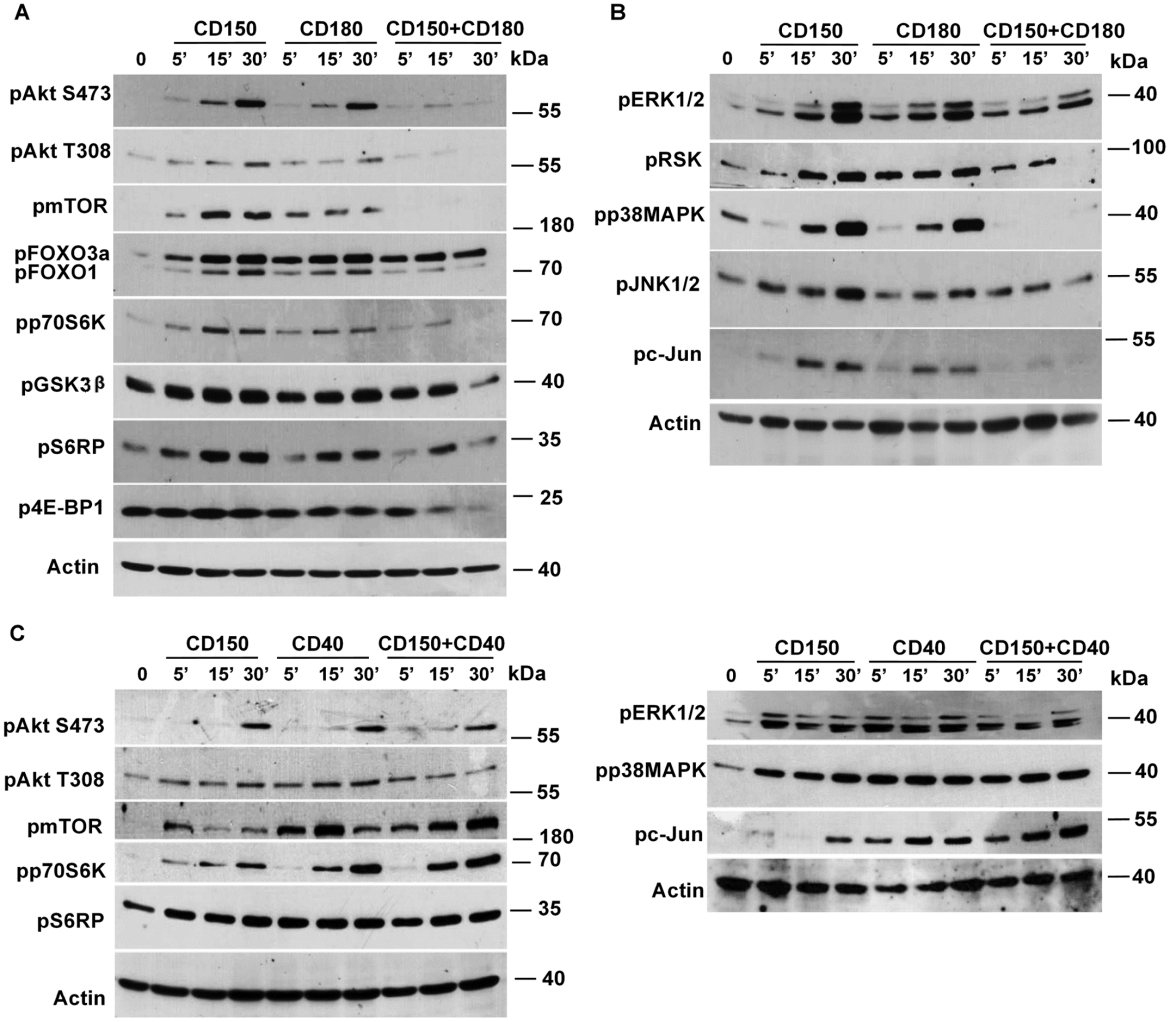
CD150 and CD180 are involved in regulation of Akt and MAPK pathways in CD150^+^CD180^+^ CLL cases. (A) Kinetic of Akt, mTOR and their downstream targets phosphorylation after CD150, CD180 and simultaneous CD150 and CD180 ligation. (B) Kinetic of MAPK pathways activation after CD150, CD180 and simultaneous CD150 and CD180 crosslinking. Ligation of CD150 or CD180 alone led to activation both Akt and MAPK pathways. However, a combination of CD150 and CD180 ligation caused a block in both Akt and MAPK signaling. (C) Coligation of CD150 and CD40 did not inhibit Akt and MAPK pathways. The results represent one of three analyzed csCD150^+^CD180^+^ CLL cases. Western blot analysis.

To find out whether csCD150^-^ and csCD150^+^ CLL B cells are different in their signaling network profiles we used two approaches: (i) comparison of signaling profiles of csCD150^-^ and csCD150^+^ CLLs and (ii) testing whether CD150 ligation trigger signaling events in CLL B cells. Since phosphorylation/dephosphorylation are key post-translational modifications that dynamically coordinate cell signaling networks, we checked the basal level of tyrosine phosphorylation and phosphorylation of serine/threonine specific motifs that are substrates for either the AMPK, Akt, PKA, PKC, or CDK kinases in CLL B cells taking into consideration cell surface CD150 expression. The csCD150^-^ and csCD150^+^ CLL B cells had different patterns of constitutive phosphorylation. In the csCD150^+^ CLL samples the basal levels of tyrosine phosphorylation generally were higher than in the csCD150^-^ samples (Fig 5A). The same pattern was observed for AMPK, Akt, PKA, PKC and CDK S/T substrates: elevated basal phosphorylation levels of S/T substrates in csCD150^+^ CLL cases compared to csCD150^-^ CLL cases. Since the mCD150 isoform with two ITSM signaling motifs in the cytoplasmic tail is predominant in CLL B cells, it may mediate signaling leading to the higher basal tyrosine and serine/threonine phosphorylation levels in csCD150^+^ CLLs. To assess this possibility we stimulated CD150^+^ CLLs with anti-CD150 mAb and compared levels of phosphorylation to unstimulated cells. CD150 ligation had no significant effect on the phosphorylation pattern either of AMPK, PKA, PKC or CDK substrates or any effect on global phosphotyrosine levels (Fig 5A). However, after CD150 ligation on csCD150^+^ CLL B cells, we detected an additional 40 kDa band in the phospho-Akt substrates (Fig 5A and 5B) suggesting that the Akt pathway was activated. Indeed, after ligation of CD150 on csCD150^+^ cells Akt was phosphorylated at S473 and T308 (Fig 6A). It should be noted that the intensity and amplitude of Akt phosphorylation at S473 were higher than at T308 after CD150 crosslinking of the CLL B cells. The peak phosphorylation of either the S473 or T308 residues occurred at 30 min after CD150 stimulation (Fig 6A). What Akt downstream targets could be phosphorylated after CD150 ligation? As evident from Fig 5A and 5B, a major Akt substrate after CD150-mediated signaling is about 38–40 kDa in size, which is about the same size as GSK3β. To test if GSK3β was phosphorylated after CD150 ligation, we used an antibody specific for phosphorylated GSK3β. Indeed, CD150 ligation increased phosphorylation of GSK3β in CLL B cells (Fig 5C). The peak of GSK3β phosphorylation corresponded to the maximum of Akt activation level at 30 min after CD150 stimulation (Fig 6A). In normal B cells CD150 ligation results in phosphorylation of the transcription factors FOXO1/FOXO3a [26]. CD150 crosslinking on CLL B cells also increased FOXO1 and FOXO3a phosphorylation levels as early as 5 min after stimulation with much higher intensity of FOXO3a phosphorylation than FOXO1 (Fig 6A). CD150-mediated signaling in CLL B cells caused phosphorylation of mTOR. Thereafter mTORC1 targeted p70S6K and its substrate ribosomal protein S6 (RPS6), but not translation inhibitor 4E-BP1 (Fig 6A).

In normal human B cells, Hodgkin’s lymphoma cell lines and the model DT40 cell line system CD150 is involved in activation not only of Akt, but also activates MAPK signaling pathways [26–28]. CD150 ligation on CLL B cells led to activation of three main groups of MAPKs—ERK1/2, JNK1/2 and p38MAPK kinases with maximum phosphorylation levels evident at 30 min after stimulation (Fig 6B). CD150-mediated ERK1/2 phosphorylation was followed by activation of its downstream target—RSK (Fig 6B). The magnitude of ERK1/2 and p38MAPK phosphorylation was higher than that for JNK1/2. CD150 crosslinking on CLL B cells resulted in activation only p54 isoform of JNK1/2. However, this was enough to phosphorylate the main JNK1/2 substrate—transcription factor c-Jun (Fig 6B). Thus, CD150 is involved in regulation of Akt and MAPK signaling in CLL B cells.

Since CD150 and CD180 showed the highest level of colocalization on the cell surface of CLL B cells, we assessed whether crosstalk between CD150 and CD180 mediated signaling pathways may happen. We performed simultaneous CD150 and CD180 coligation on CLL B cells in comparison with CD150 or CD180 crosslinking alone. The kinetics of CD150 and CD180 mediated Akt and MAPK activation was similar (Fig 6A and 6B). However, CD150 and CD180 coligation showed strong dephosphorylation of Akt at both the S473 and T308 residues with a maximum reduction of Akt phosphorylation at 30 min after stimulation (Fig 6A). In line with this Akt substrates were dephosphorylated after coligation of these receptors (Panel A in S1 Fig). Furthermore, mTOR was not phosphorylated that partially contribute to lower phosphorylation of p70S6K, RPS6 and 4E-BP1 (Fig 6A). Phospho-GSK3β declined even below its basal phosphorylation level at 30 min after CD150 and CD180 coligation (Fig 6A). However, attenuation of Akt activation via simultaneous CD150+CD180 coligation did not completely inhibit either FOXO1 or FOXO3a phosphorylation (Fig 6A). Both c-Jun and RSK phosphorylation levels were significantly reduced after CD150+CD180 stimulation even though coligation of these receptors did not have a strong impact on JNK1/2 or ERK1/2 dephosphorylation (Fig 6B). CD150 and CD180 coligation also led to apparent attenuation of p38MAPK signaling in CLL B cells (Fig 6B). At the same time coligation of CD150 and CD40, which are not colocalized, did not result in Akt and MAPK pathways inhibition (Fig 6C). Coligation of these receptors did not have additive or synergistic effect on kinetics and amplitude of Akt and MAPK networks activation, except pp70S6K and pc-Jun (Fig 6C). Taking together, CD150 and CD180 receptors alone are linked to activation of Akt and MAPK signal transduction pathways in CLL B cells. However, simultaneous CD150 and CD180 coligation resulted in inactivation of parts of the Akt and MAPK signaling pathways.

## Discussion

CD150/SLAMF1 is ubiquitously expressed on normal B lineage cells and is detectable as early as the pro-B cell stage; it is reduced in expression on immature bone marrow B cells and then increases toward plasma cell stage [28,29,30]. Numerous data indicate that CD150 is aberrantly expressed on the surface of malignant B cells. Neoplastic B cells that reflect CD150^+^ normal B cell counterparts do not always express cell surface CD150. CD150^-^ B-cell malignancies include pre-B acute lymphoblastic leukemia, small lymphocytic lymphoma, sporadic Burkitt’s lymphoma, germinal center subtype of diffuse large B-cell lymphoma, lymphoplasmacytic lymphoma, and primary cutaneous marginal zone B-cell lymphoma [27,31–33]. In several types of B-cell malignancies CD150 surface expression is much lower than that on their normal B-cell counterparts. For example, primary mantle cell lymphoma has very low CD150 expression [33]. Even though CD150 is expressed at its highest levels on normal plasma cells, primary multiple myeloma (MM) cases have low CD150 expression levels. Moreover, the number of CD150^+^ neoplastic B cells is dramatically decreased in relapse MM cases in comparison to newly diagnosed MM cases [34]. In malignant B-cell lines *in vitro* CD150 protein expression is limited and not always correlates with CD150 mRNA level [35]. These findings suggest that malignant B cells may try to escape/reduce CD150 cell surface expression.

What might be the role of CD150 in the pathobiology of malignant B cells? Our studies and others [6,17,18] have revealed significant heterogeneity of CD150 surface expression in CLL cases. In addition, we found that even CD150 surface negative CLL B cells express CD150 protein that is retained in the cytoplasm (Fig 2B and 2C). The pattern of CD150 localization in cytoplasmic compartments (ER, Golgi and endosomes, but not in lysosomes) implies that this receptor is normally synthesised in the ER, undergoes processing in Golgi complex and is not subject to degradation. The fact that intracellular CD150 is present in early endosomes may reflect CD150 recycling between the cell surface and cytoplasmic compartments. Cell-cell interactions between csCD150^+^ CLL B cells, T cells or ligation of CD150 by a soluble CD150 isoform in lymphoid tissues may initiate CD150 internalization that results in loss of csCD150 expression on recirculating peripheral blood CLL B cells. A similar mechanism has been described in CLL for BCR expression and loss [9]. Moreover, the fact that CD150 is involved in phagosome formation [15] and a recently described mechanism for CD150-dependent measles virus internalization [36] also support the idea that CD150 is recycling in csCD150^-^ cases.

The levels of cell surface CD180 are higher in CLL cases with mutated *IGHV*, making CD180, like CD150, a potential surrogate prognostic marker of CLL outcome [21]. CD180 ligation can modulate BCR signaling in CLL B cells, preventing pro-survival signals and inducing apoptosis [37]. We found that CD150 cell surface expression positively correlated with CD180 expression on CLL B cells. Moreover, these receptors were colocalized on CLL B lymphocytes (Fig 1B). Similar to CD150, in CD180 surface negative CLLs CD180 was detected in the cytoplasm. However, the subcellular localization of CD180 was different from CD150. Preferential CD180 colocalization with an early endosome marker implies possible CD180 internalization in csCD180^-^ CLL cases, especially since CD180 is known to internalize after stimulation with mAbs [23].

Why CD150 is not expressed on all neoplastic B cell CLL cases and retained in cytoplasm? One possibility was that the csCD150^-^ malignant B cells express a CD150 isoform that lacks a transmembrane domain. Indeed, our analysis of CD150 isoform expression showed significantly elevated expression of the soluble sCD150 in vast majority the CLLs we examined (Fig 4C). Moreover, we found that it is secreted by CLL B cells (Fig 4D). Since CD150 is a ligand for itself [38], the secreted CD150 isoform may possibly be involved in constitutive activation through the CD150 receptor. The biological significance of sCD150 expression and secretion in CLL needs further investigation. Another possibility for exclusive intracellular localisation of CD150 in csCD150^-^ CLLs is that CD150 may lack a leader peptide. Given that only a minor portion of CD150 was not glycosylated and was represented by the 40 kDa band (Fig 2B), this possibility is unlikely.

The mCD150 isoform with two ITSM signaling motifs is a predominant CD150 isoform in CLL B cells (Fig 4A), and thus, would be expected to have signaling properties. Indeed, in neoplastic B cells the basal phosphorylation levels of tyrosine and AMPK, Akt, PKA, PKC, and CDK S/T substrates were higher in csCD150^+^ CLL cases compared to csCD150^-^ CLL cases. However, only the phosphorylation of Akt and Akt substrates were CD150 specific. Our data suggest that CD150 and CD180 are both involved in overlapping cell signaling pathways that regulate protein synthesis, cell survival, and possibly cell proliferation. These receptors affect protein synthesis in CLL B cells via Akt-mTORC1 axis activating p70S6K (Fig 5) that is involved in assembling of initiation and elongation translation complex and ribosomal biogenesis [39,40]. While p70S6K can phosphorylate RPS6 at all sites, S235/236 is a specific target for RSK [41]. Since the level of CD150 and CD180 mediated RPS6 phosphorylation was much higher that can be expected from the level of p70S6K activation (Fig 5D), it is possible that S235/236 in RPS6 may be also phosphorylated by RSK. Indeed, ligation of these receptors in addition to Akt-mTORC1 pathways activated the ERK1/2-RSK axis (Fig 5E). Moreover, cross-talk between Akt and ERK1/2-RSK pathways in targeting mTORC1 may also amplify translational events [42]. It is clear that both CD150 and CD180 receptor pathways can impinge on the translation machinery through mTOR independent as well as mTOR dependent manner.

Akt-mediated inactivation of GSK3β and FOXO after CD150 and CD180 stimulation suggests a pro-survival role for these receptors in CLL B cells pathobiology, while cross-talk between CD180 and BCR can redirect signaling from pro-survival to pro-apoptotic pathways [37]. Upregulation of phospho-ERK1/2 level may affect not only translational machinery, but also promote malignant B cell proliferation and viability [43]. Activation of the ERK1/2 pathway is a key downstream target of BCR signaling and is associated with increased CLL B cells survival, resistance to chemotherapy and upregulation of chemokine secretion [44]. However, in *IGHV*-mutated CLL cases elevated pERK1/2 level is a feature of anergic B cells [9]. The role of p38MAPK and JNK1/2 pathways in CLL pathogenesis is controversial. From one side, p38MAPK and JNK1/2 are also involved in BCR signaling [45]. However, Bologna et al. showed that CD150-mediated JNK1/2 activation in CLL B cells is required for autophagosome formation via BCL2 phosphorylation and consequent Beclin1 dissociation [18].

There is a disagreement between favourable prognosis for CD150^+^ and CD180^+^ CLLs and activation of pro-survival pathways via these receptors. Unexpectedly, simultaneous coligation of the CD150 and CD180 receptors on CLL B cells led to cross-inhibition of the Akt and MAPK pathways (Fig 6). This effect does not seem to be simply due to a physical block of e.g., receptor internalization since the inhibition was selective and did not affect phosphorylation of FOXO3a (Fig 6). The decrease of Akt kinase activity in turn had the strongest effect on mTORC1 pathways that regulate translational machinery, rather than on FOXO1/3a or GSK3β. CD150 and CD180 mediated Akt and MAPK pathways inhibition seems to be receptor specific since CD150 and CD40 coligation did not lead to similar effect.

The mechanisms that underlie the inhibition of the Akt and MAPK pathways after CD150 and CD180 coligation on CLL B cells are not clear. Hypothetically, simultaneous crosslinking of these receptors may activate phosphatases that selectively negatively regulate stages of the Akt and MAPK pathways. In B cells CD150 via its ITSM motifs associates with the SH2-containing protein tyrosine phosphatase SHP-2 and the inositol phosphatase SHIP. In addition CD150 coprecipitates with the receptor tyrosine phosphatase CD45 [46,47]. It is unlikely that SHIP is involved in reduction of Akt and MAPK pathways after CD150 and CD180 coligation on CLLs since we found no correlation between CD150 and SHIP expression (Panels B and C in S1 Fig). Despite the presence of a conserved tyrosine residue within the CD180 cytoplasmic domain and its association with PIM-1L kinase, the signaling properties of CD180 appear mainly to depend on interactions with CD19 [24,48]. In B cells CD150 and CD19 are colocalized (Fig 1B), coprecipitate with and are substrates for Lyn that creates phosphorylated binding sites for the p85 regulatory subunit of PI3 kinase, phosphatases and other SH2-containing components of signaling pathways [26,27,46,49]. Coligation of CD150 and CD180/CD19 may rapidly turn-on a Lyn-SHP-1/2 inactivation loop that results in partial inhibition of Akt-mTOR and MAPK networks. This hypothesis is supported by three types of responses after CD150 and CD180 coligation: reduced phosphorylation (ERK1/2, JNK1/2; FOXO1, c-Jun), dephosphorylation at 15–30 min (Akt, RSK, p70S6K, S6RP; 4E-BP, GSK3β} and complete blocking of phosphorylation (p38MAPK and mTOR) (Fig 6). Thus, simultaneous CD150 and CD180 ligation more likely inhibit mRNA translation than induce pro-apoptotic effect in CLL B cells. Since MAPK pathways eventually target TF [50], full attenuation of p38MAPK and c-Jun kinases together with partial silencing of ERK1/2 after CD150 and CD180 simultaneous ligation could result in decreased transcriptional activity in CLL B cells (Fig 6B). Given that mTORC1 is involved in the process of autophagy [40,51], mTOR dephosphorylation after concomitant CD150 and CD180 signaling provide an additional link to autophagy regulation.

Our data suggest that CD150 or CD180 expression and activation alone is not sufficient to ensure the features of favourable clinical outcome in CLL patients. We hypothesize that in more than 50% of CLL cases where CD150 and CD180 are coexpressed simultaneous ligation of these receptors on malignant CLL B cells may result in inhibition of pro-survival Akt and MAPK signaling pathways. Could such coligation occur *in vivo* in CLL patients? CD180 is a pattern recognizing receptor [24]. Since CD150 is a bacterial sensor, receptor for hemagglutinins of morbilliviruses and a self-ligand, it also could be considered as a pattern recognizing receptor. Common in CLL microbial and autoantigens, as well as apoptosis evoked neo-antigens may create pattern platforms for simultaneous ligation of CD150 and CD180. Further studies of CD150 and CD180 double positive CLLs will reveal the clinical significance of these receptors and their coexpression in CLLs.

All mentioned above suggest that combination of signals via CD150 and CD180 may be a restraining factor for neoplastic CLL B cells propagation. Therefore, malignant CLL B cells try to escape simultaneous cell surface CD150 and CD180 expression, since combined ligation of these receptors leads to blocking of pro-survival pathways. Our studies not only broaden the knowledge about CD150 and CD180 involvement in CLL pathobiology, but also suggest additional receptor-directed approaches for improvement of CLL patients’ survival and quality of life.

## Supporting information

S1 Table Clinicopathological details of CLL patients. (DOC)

S2 Table The primers sequence used in study. (DOC)

S3 Table Immunophenotyping of CLL malignant B cells. (DOC)

S1 Fig Akt substrates phosphorylation and SHIP-1 protein expression in CLL cases. (A) Downregulation of Akt substrates phosphorylation after CD150 and CD180 coligation, western blot analysis. (B) Western blot analysis of SHIP-1 expression in CLL samples taking into consideration cell surface (cs) CD150 expression. (C) Densitometry analysis of SHIP-1 protein expression level in CLL cases. Results were normalized to the actin expression level.
(TIF)

## References

[1] Fabbri G, Dalla-Favera R. The molecular pathogenesis of chronic lymphocytic leukaemia. Nat Rev Cancer. 2016;16(3):145–62. doi: 10.1038/nrc.2016.8 26911189 10.1038/nrc.2016.8

[2] Landau DA, Tausch E, Taylor-Weiner AN, Stewart C, Reiter JG, Bahlo J, et al Mutations driving CLL and their evolution in progression and relapse. Nature. 2015;526(7574):525–30. doi: 10.1038/nature15395 26466571 10.1038/nature15395 PMC4815041

[3] Oakes CC, Seifert M, Assenov Y, Gu L, Przekopowitz M, Ruppert AS, et al DNA methylation dynamics during B cell maturation underlie a continuum of disease phenotypes in chronic lymphocytic leukemia. Nat Genet. 2016;48(3):253–64. doi: 10.1038/ng.3488 26780610 10.1038/ng.3488 PMC4963005

[4] Guieze R, Wu CJ. Genomic and epigenomic heterogeneity in chronic lymphocytic leukemia. Blood. 2015;126(4):445–53. doi: 10.1182/blood-2015-02-585042 26065654 10.1182/blood-2015-02-585042 PMC4513249

[5] Huang PY, Best OG, Almazi JG, Belov L, Davis ZA, Majid A, et al Cell surface phenotype profiles distinguish stable and progressive chronic lymphocytic leukemia. Leuk Lymphoma. 2014;55(9):2085–92. doi: 10.3109/10428194.2013.867486 24289109 10.3109/10428194.2013.867486

[6] Zucchetto A, Cattarossi I, Nanni P, Zaina E, Prato G, Gilestro M, et al Cluster analysis of immunophenotypic data: the example of chronic lymphocytic leukemia. Immunol Lett. 2011;134(2):137–44. doi: 10.1016/j.imlet.2010.09.017 20923685 10.1016/j.imlet.2010.09.017

[7] Wiestner A. The role of B-cell receptor inhibitors in the treatment of patients with chronic lymphocytic leukemia. Haematologica. 2015;100(12):1495–507. doi: 10.3324/haematol.2014.119123 26628631 10.3324/haematol.2014.119123 PMC4666325

[8] Woyach JA, Johnson AJ. Targeted therapies in CLL: mechanisms of resistance and strategies for management. Blood. 2015;126(4):471–7. doi: 10.1182/blood-2015-03-585075 26065659 10.1182/blood-2015-03-585075 PMC4513250

[9] Packham G, Krysov S, Allen A, Savelyeva N, Steele AJ, Forconi F, et al The outcome of B-cell receptor signaling in chronic lymphocytic leukemia: proliferation or anergy. Haematologica. 2014;99(7):1138–48. doi: 10.3324/haematol.2013.098384 24986876 10.3324/haematol.2013.098384 PMC4077074

[10] Zhang S, Kipps TJ. The pathogenesis of chronic lymphocytic leukemia. Annu Rev Pathol. 2014;9:103–18. doi: 10.1146/annurev-pathol-020712-163955 23987584 10.1146/annurev-pathol-020712-163955 PMC4144790

[11] Sidorenko SP, Clark EA. Characterization of a cell surface glycoprotein IPO-3, expressed on activated human B and T lymphocytes. J Immunol. 1993;151(9):4614–24. 8409422

[12] Cocks BG, Chang CC, Carballido JM, Yssel H, de Vries JE, Aversa G. A novel receptor involved in T-cell activation. Nature. 1995;376(6537):260–3. doi: 10.1038/376260a0 7617038 10.1038/376260a0

[13] Sidorenko SP, Clark EA. The dual-function CD150 receptor subfamily: the viral attraction. Nat Immunol. 2003;4(1):19–24. doi: 10.1038/ni0103-19 12496974 10.1038/ni0103-19

[14] Sintes J, Engel P. SLAM (CD150) is a multitasking immunoreceptor: from cosignalling to bacterial recognition. Immunol Cell Biol. 2011;89(2):161–3. doi: 10.1038/icb.2010.145 21102539 10.1038/icb.2010.145

[15] Berger SB, Romero X, Ma C, Wang G, Faubion WA, Liao G, et al SLAM is a microbial sensor that regulates bacterial phagosome functions in macrophages. Nat Immunol. 2010;11(10):920–7. doi: 10.1038/ni.1931 20818396 10.1038/ni.1931 PMC3338319

[16] Wu N, Veillette A. SLAM family receptors in normal immunity and immune pathologies. Curr Opin Immunol. 2016;38:45–51. doi: 10.1016/j.coi.2015.11.003 26682762 10.1016/j.coi.2015.11.003

[17] Schweighofer CD, Coombes KR, Barron LL, Diao L, Newman RJ, Ferrajoli A, et al A two-gene signature, SKI and SLAMF1, predicts time-to-treatment in previously untreated patients with chronic lymphocytic leukemia. PLoS One. 2011;6(12):e28277 doi: 10.1371/journal.pone.0028277 22194822 10.1371/journal.pone.0028277 PMC3237436

[18] Bologna C, Buonincontri R, Serra S, Vaisitti T, Audrito V, Brusa D, et al SLAMF1 regulation of chemotaxis and autophagy determines CLL patient response. J Clin Invest. 2016;126(1):181–94. doi: 10.1172/JCI83013 26619119 10.1172/JCI83013 PMC4701571

[19] Punnonen J, Cocks BG, Carballido JM, Bennett B, Peterson D, Aversa G, et al Soluble and membrane-bound forms of signaling lymphocytic activation molecule (SLAM) induce proliferation and Ig synthesis by activated human B lymphocytes. J Exp Med. 1997;185(6):993–1004. 9091591 10.1084/jem.185.6.993 PMC2196230

[20] Romanets-Korbut O, Najakshin AM, Yurchenko M, Malysheva TA, Kovalevska L, Shlapatska LM, et al Expression of CD150 in tumors of the central nervous system: identification of a novel isoform. PLoS One. 2015;10(2):e0118302 doi: 10.1371/journal.pone.0118302 25710480 10.1371/journal.pone.0118302 PMC4339833

[21] Porakishvili N, Kulikova N, Jewell AP, Youinou PY, Yong K, Nathwani A, et al Differential expression of CD 180 and IgM by B-cell chronic lymphocytic leukaemia cells using mutated and unmutated immunoglobulin VH genes. Br J Haematol. 2005;131(3):313–9. doi: 10.1111/j.1365-2141.2005.05775.x 16225650 10.1111/j.1365-2141.2005.05775.x

[22] Chaplin JW, Kasahara S, Clark EA, Ledbetter JA. Anti-CD180 (RP105) activates B cells to rapidly produce polyclonal Ig via a T cell and MyD88-independent pathway. J Immunol. 2011;187(8):4199–209. doi: 10.4049/jimmunol.1100198 21918197 10.4049/jimmunol.1100198 PMC3394853

[23] Chaplin JW, Chappell CP, Clark EA. Targeting antigens to CD180 rapidly induces antigen-specific IgG, affinity maturation, and immunological memory. J Exp Med. 2013;210(10):2135–46. doi: 10.1084/jem.20130188 24019553 10.1084/jem.20130188 PMC3782047

[24] Schultz TE, Blumenthal A. The RP105/MD-1 complex: molecular signaling mechanisms and pathophysiological implications. J Leukoc Biol. 2017;101(1):183–92. doi: 10.1189/jlb.2VMR1215-582R 27067450 10.1189/jlb.2VMR1215-582R

[25] Shlapatska LM, Kovalevska LM, Gordiienko IM, Sidorenko SP. Intrinsic defect in B-lymphoblastoid cell lines from patients with X-linked lymphoproliferative disease type 1. II. receptor-mediated Akt/PKB and ERK1/2 activation and transcription factors expression profile. Exp Oncol. 2014;36(3):162–9. 25265348

[26] Yurchenko M, Shlapatska LM, Romanets OL, Ganshevskiy D, Kashuba E, Zamoshnikova A, et al CD150-mediated Akt signalling pathway in normal and malignant B cells. Exp Oncol. 2011;33(1):9–18. 21423089

[27] Mikhalap SV, Shlapatska LM, Yurchenko OV, Yurchenko MY, Berdova GG, Nichols KE, et al The adaptor protein SH2D1A regulates signaling through CD150 (SLAM) in B cells. Blood. 2004;104(13):4063–70. doi: 10.1182/blood-2004-04-1273 15315965 10.1182/blood-2004-04-1273

[28] Yurchenko MY, Kovalevska LM, Shlapatska LM, Berdova GG, Clark EA, Sidorenko SP. CD150 regulates JNK1/2 activation in normal and Hodgkin's lymphoma B cells. Immunol Cell Biol. 2010;88(5):565–74. doi: 10.1038/icb.2010.14 20231852 10.1038/icb.2010.14

[29] De Salort J, Sintes J, Llinas L, Matesanz-Isabel J, Engel P. Expression of SLAM (CD150) cell-surface receptors on human B-cell subsets: from pro-B to plasma cells. Immunol Lett. 2011;134(2):129–36. doi: 10.1016/j.imlet.2010.09.021 20933013 10.1016/j.imlet.2010.09.021

[30] Rodriguez-Bayona B, Ramos-Amaya A, Brieva JA. Differential expression of SLAMS and other modulatory molecules by human plasma cells during normal maturation. Immunol Lett. 2011;134(2):122–8. doi: 10.1016/j.imlet.2010.09.015 20923684 10.1016/j.imlet.2010.09.015

[31] Sidorenko SP, Vetrova EP, Yurchenko OV, Berdova AG, Shlapatskaya LN, Gluzman DF. Monoclonal antibodies of IPO series against B cell differentiation antigens in leukemia and lymphoma immunophenotyping. Neoplasma. 1992;39(1):3–9. 1528302

[32] Fanoni D, Tavecchio S, Recalcati S, Balice Y, Venegoni L, Fiorani R, et al New monoclonal antibodies against B-cell antigens: possible new strategies for diagnosis of primary cutaneous B-cell lymphomas. Immunol Lett. 2011;134(2):157–60. doi: 10.1016/j.imlet.2010.09.022 20951741 10.1016/j.imlet.2010.09.022

[33] Yurchenko O.V., Shlapatska L.M., Skryma M.R., Berdova G.G., Kovalevska L.M., Tarasova T.A., et al Immunohistochemical studies of CD150 expression in some human tumors. Experimental Oncology. 2003;25(3):186–90.

[34] Muccio VE, Saraci E, Gilestro M, Gattei V, Zucchetto A, Astolfi M, et al Multiple myeloma: New surface antigens for the characterization of plasma cells in the era of novel agents. Cytometry B Clin Cytom. 2016;90(1):81–90. doi: 10.1002/cyto.b.21279 26287276 10.1002/cyto.b.21279

[35] Gordiienko IM, Shlapatska LM, Kovalevska LM, Sidorenko SP. Differential expression of CD150/SLAMF1 in normal and malignant B cells on the different stages of maturation. Exp Oncol. 2016;38(2):101–7. 27356578

[36] Goncalves-Carneiro D, McKeating JA, Bailey D. The measles virus receptor SLAMF1 can mediate particle endocytosis. J Virol. 2017;91(7). doi: 10.1128/JVI.02255-16 28100610 10.1128/JVI.02255-16 PMC5355598

[37] Porakishvili N, Vispute K, Steele AJ, Rajakaruna N, Kulikova N, Tsertsvadze T, et al Rewiring of sIgM-mediated intracellular signaling through the CD180 Toll-like receptor. Mol Med. 2015; 21(1):46–57. 10.2119/molmed.2014.00265 PMC4461575 25611435

[38] Mavaddat N, Mason DW, Atkinson PD, Evans EJ, Gilbert RJ, Stuart DI, et al Signaling lymphocytic activation molecule (CDw150) is homophilic but self-associates with very low affinity. J Biol Chem. 2000;275(36):28100–9. doi: 10.1074/jbc.M004117200 10831600 10.1074/jbc.M004117200

[39] Bhaskar PT, Hay N. The two TORCs and Akt. Dev Cell. 2007;12(4):487–502. doi: 10.1016/j.devcel.2007.03.020 17419990 10.1016/j.devcel.2007.03.020

[40] Zoncu R, Efeyan A, Sabatini DM. mTOR: from growth signal integration to cancer, diabetes and ageing. Nat Rev Mol Cell Biol. 2011;12(1):21–35. doi: 10.1038/nrm3025 21157483 10.1038/nrm3025 PMC3390257

[41] Roux PP, Shahbazian D, Vu H, Holz MK, Cohen MS, Taunton J, et al RAS/ERK signaling promotes site-specific ribosomal protein S6 phosphorylation via RSK and stimulates cap-dependent translation. J Biol Chem. 2007;282(19):14056–64. doi: 10.1074/jbc.M700906200 17360704 10.1074/jbc.M700906200 PMC3618456

[42] Mendoza MC, Er EE, Blenis J. The Ras-ERK and PI3K-mTOR pathways: cross-talk and compensation. Trends Biochem Sci. 2011;36(6):320–8. doi: 10.1016/j.tibs.2011.03.006 21531565 10.1016/j.tibs.2011.03.006 PMC3112285

[43] Zhang W, Liu HT. MAPK signal pathways in the regulation of cell proliferation in mammalian cells. Cell Res. 2002;12(1):9–18. doi: 10.1038/sj.cr.7290105 11942415 10.1038/sj.cr.7290105

[44] Ten Hacken E, Sivina M, Kim E, O'Brien S, Wierda WG, Ferrajoli A, et al Functional differences between IgM and IgD signaling in chronic lymphocytic leukemia. J Immunol. 2016; 197 (6) 2522–31. doi: 10.4049/jimmunol.1600915 27534555 10.4049/jimmunol.1600915 PMC5010921

[45] Guarini A, Chiaretti S, Tavolaro S, Maggio R, Peragine N, Citarella F, et al BCR ligation induced by IgM stimulation results in gene expression and functional changes only in IgV H unmutated chronic lymphocytic leukemia (CLL) cells. Blood. 2008;112(3):782–92. doi: 10.1182/blood-2007-12-127688 18487510 10.1182/blood-2007-12-127688

[46] Mikhalap SV, Shlapatska LM, Berdova AG, Law CL, Clark EA, Sidorenko SP. CDw150 associates with src-homology 2-containing inositol phosphatase and modulates CD95-mediated apoptosis. J Immunol. 1999;162(10):5719–27. 10229804

[47] Shlapatska LM, Mikhalap SV, Berdova AG, Zelensky OM, Yun TJ, Nichols KE, et al CD150 association with either the SH2-containing inositol phosphatase or the SH2-containing protein tyrosine phosphatase is regulated by the adaptor protein SH2D1A. J Immunol. 2001;166(9):5480–7. 11313386 10.4049/jimmunol.166.9.5480

[48] Ingley E. Functions of the Lyn tyrosine kinase in health and disease. Cell Commun Signal. 2012;10(1):10–21. 22805580 10.1186/1478-811X-10-21 PMC3464935

[49] Somani AK, Yuen K, Xu F, Zhang J, Branch DR, Siminovitch KA. The SH2 domain containing tyrosine phosphatase-1 down-regulates activation of Lyn and Lyn-induced tyrosine phosphorylation of the CD19 receptor in B cells. J Biol Chem. 2001;276(3):1938–44. doi: 10.1074/jbc.M006820200 11042209 10.1074/jbc.M006820200

[50] Kim EK, Choi EJ. Pathological roles of MAPK signaling pathways in human diseases. Biochim Biophys Acta. 2010;1802(4):396–405. doi: 10.1016/j.bbadis.2009.12.009 20079433 10.1016/j.bbadis.2009.12.009

[51] Neufeld TP. TOR-dependent control of autophagy: biting the hand that feeds. Curr Opin Cell Biol. 2010;22(2):157–68. doi: 10.1016/j.ceb.2009.11.005 20006481 10.1016/j.ceb.2009.11.005 PMC2854204

