# Supplementary material for: The interplay of CD150 and CD180 receptor pathways contribute to the pathobiology of chronic lymphocytic leukemia B cells by selective inhibition of Akt and MAPK signaling
Source: PLoS One. 2017 Oct 5;12(10):e0185940. doi: 10.1371/journal.pone.0185940 (PMC5628907; doi:10.1371/journal.pone.0185940)
Supplement: S1 Table — (DOC) [file pone.0185940.s001.doc]

**S1 Table. Clinicopathological details of CLL patients**

| **Clinicopathological characteristics** | | | **Patient N (%)** |
| --- | --- | --- | --- |
| Age | ≤60 | | 27 (40%) |
|  | >60 | | 40 (60%) |
| Gender | Male | | 43 (64.8%) |
|  | Female | | 24 (35.2%) |
| Anemia | | | 20 (30%) |
| Trombocytopenia | | | 17 (25%) |
| WBC count, x10^9^, (median, range) | | | 86 (14.4-390) |
| Lymphadenopathy | | | 46 (68%) |
| Splenomegalia | | | 36 (54%) |
| Hepatomegalia | | | 42 (63%) |
| CD38 | | ≤30% | 38 (56,3%) |
|  |  | >30% | 29 (43,7%) |
