# Supplementary material for: The interplay of CD150 and CD180 receptor pathways contribute to the pathobiology of chronic lymphocytic leukemia B cells by selective inhibition of Akt and MAPK signaling
Source: PLoS One. 2017 Oct 5;12(10):e0185940. doi: 10.1371/journal.pone.0185940 (PMC5628907; doi:10.1371/journal.pone.0185940)
Supplement: S2 Table — (DOC) [file pone.0185940.s002.doc]

**S2 Table. The primers sequence** used in study

| ***Gene*** | **Forward primer**  **5’→3’** | **Reverse primer**  **5’→3’** |
| --- | --- | --- |
| *TBP* | ccactcacagactctcacaac | ctgcggtacaatcccagaact |
| *ExtrCD150* | aggccctccacgttatcta | gcaaaagcgctgaactga |
| *Cyt-m CD150* | gtgtatgctgggctgttagg | agaggtaaaacgaaccattacca |
| *Cyt-n CD150* | tgagaagaagagccaccttga | ggttcgttttaccatgggaag |
| *sCD150* | agacccctcaggtaaaacg | tctggacttgggcatagatcg |
