# Supplementary material for: The interplay of CD150 and CD180 receptor pathways contribute to the pathobiology of chronic lymphocytic leukemia B cells by selective inhibition of Akt and MAPK signaling
Source: PLoS One. 2017 Oct 5;12(10):e0185940. doi: 10.1371/journal.pone.0185940 (PMC5628907; doi:10.1371/journal.pone.0185940)
Supplement: S3 Table — (DOC) [file pone.0185940.s003.doc]

**S3 Table. Immunophenotyping of CLL malignant B cells**

| **CD marker** | **% of positive CLL cases** | **% of PC**  **Median (range)** | **GeoMean antigen/**  **GeoMean isotype control**  **Median (range)** |
| --- | --- | --- | --- |
| CD5 | 100 | 65(8-94) | 3,1(1,2-7,6) |
| CD19 | 100 | 82 (62-95) | 7,1 (4,9 – 17,3) |
| CD20 | 91 | 55(0-94) | 2,4 (1-8,9) |
| CD22 | 48 | 4 (0-87) | 1,1 (1,0-4,5) |
| CD23 | 100 | 60 (43-89) | 3,0 (2,0-5,5) |
| CD43 | 100 | 80 (69-92) | 4,9(5,2-7,4) |
| CD37 | 100,0 | 81 (47-95) | 5,1 (2,1-13,0) |
| CD38 | 56,3 | 13 (0-87) | 1,4 (1,0-5,2) |
| CD40 | 97,0 | 75 (0-95) | 3,1 (1,0-23,0) |
| CD48 | 100,0 | 90 (42-100) | 8,5 (2,5-54,4) |
| CD95 | 71,2 | 27 (0-72) | 1,4 (1,0-5,5) |
| CD150 | 71,6 | 16 (0-93) | 1,5 (1,0-6,9) |
| CD180 | 69,2 | 14,5 (0-79) | 1,3 (1,0-4,1) |
