# Supplementary figures and images for: The interplay of CD150 and CD180 receptor pathways contribute to the pathobiology of chronic lymphocytic leukemia B cells by selective inhibition of Akt and MAPK signaling
Source: PLoS One. 2017 Oct 5;12(10):e0185940. doi: 10.1371/journal.pone.0185940 (PMC5628907; doi:10.1371/journal.pone.0185940)

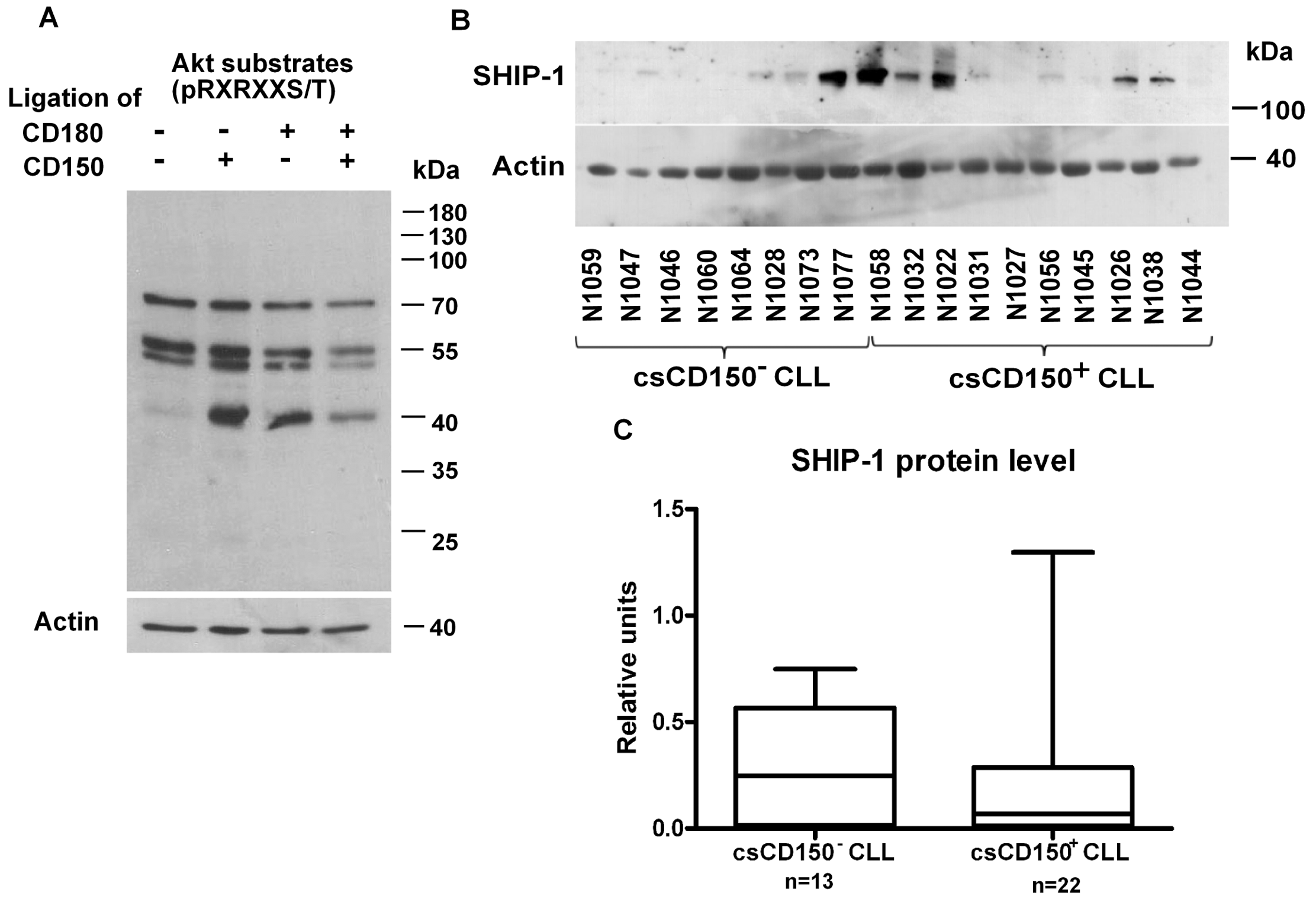

Supplement: S1 Fig — (A) Downregulation of Akt substrates phosphorylation after CD150 and CD180 coligation, western blot analysis. (B) Western blot analysis of SHIP-1 expression in CLL samples taking into consideration cell surface (cs) CD150 expression. (C) Densitometry analysis of SHIP-1 protein expression level in CLL cases. Results were normalized to the actin expression level. (TIF) [file pone.0185940.s004.tif]
